# Annexin A2 Expression in Aerogenous Metastasis of Pulmonary Invasive Mucinous Adenocarcinoma: A Case Report including Immunohistochemical Analysis

**DOI:** 10.1155/2019/5064852

**Published:** 2019-08-07

**Authors:** Kazumori Arai, Masahide Hirose

**Affiliations:** ^1^Department of Pathology, Shizuoka General Hospital, 4-27-1 Kitaando, Aoi-ku, Shizuoka 420-0881, Japan; ^2^Department of Thoracic Surgery, Shizuoka General Hospital, 4-27-1 Kitaando, Aoi-ku, Shizuoka 420-0881, Japan

## Abstract

Aerogenous metastasis (AM) is a form of lung cancer that spreads in a unique fashion, but its mechanisms are still unclear. Annexin A2 (ANX A2), a membrane-binding protein, promotes cancer invasion and is involved in cell adhesion and polarity. The relationship between ANX A2 and cancers with poor stromal invasion capacity has not been studied. We immunohistochemically analyzed ANX A2 expression in AM observed in a patient with pulmonary invasive mucinous adenocarcinoma. In the primary site, ANX A2 immunopositivity on the cell-cell borders weakened as tumor cells projected and separated into alveolar spaces. In AM, tumor cell aggregates with ANX A2 immunopositivity near the surface and within the cytoplasm attached to alveolar epithelial cells, then engulfed them and formed a protrusion. As tumor cell aggregates adhered to the alveolar wall and formed a single layer, cytoplasmic ANX A2-positive products accumulated in the lateral sides of the tumor cells and exhibited distinct membranous positivity. These results indicated that ANX A2 near the tumor cell surface was related to alveolar wall attachment. Furthermore, the translocation of cytoplasmic ANX A2 to cell-cell borders changed cell morphology, adhesion, and polarity restoration.

## 1. Introduction

Aerogenous metastasis (AM) is a characteristic form of lung cancer progression [1]. AM is defined as a noncontiguous spread to lung parenchyma, via the airways from the primary site [1]. According to the 2015 WHO classification [2], spread through air spaces (STAS) is the fourth category that defines lung adenocarcinoma (AC) invasion. STAS is considered the origin of AM [3, 4]. AM significantly increases recurrence rates and is regarded as a poor prognostic factor [1–4]. However, its pathogenic mechanisms remain unclear.

Annexin A2 (ANX A2) belongs to a family of Ca^2+^/membrane-binding proteins [5]. This protein is involved in actin cytoskeleton dynamics, cell adhesion, cell polarity, and phagocytosis within the cytoplasm and the plasma membranes of various cells [5–8]; however, its functions have not been elucidated fully. ANX A2 is also expressed in various cancers and promotes cancer cell invasion [9–11]. In non-small-cell lung cancer, ANX A2 expression is correlated with poor pathological parameters and regarded as an indicator of worse prognosis [12, 13]. ANX A2 is a novel serum biomarker for lung cancer [14], and its involvement in chemotherapy resistance in lung cancer has attracted attention [13]. However, cancers with poor stromal invasion capacity have not been researched in terms of their relationship to ANX A2.

Invasive mucinous adenocarcinoma (IMAC) of the lung, formerly referred to as mucinous bronchioloalveolar carcinoma, is a rare type of lung AC [2, 3] that frequently exhibits STAS/AM [1, 3]. We hypothesized that ANX A2 is also involved in AM, which is a unique from of cancer progression, unlike stromal invasion. In this report, we immunohistochemically examined the expression of ANX A2 in a case of IMAC.

## 2. Case Presentation

### 2.1. Case History

**Figure 1:**
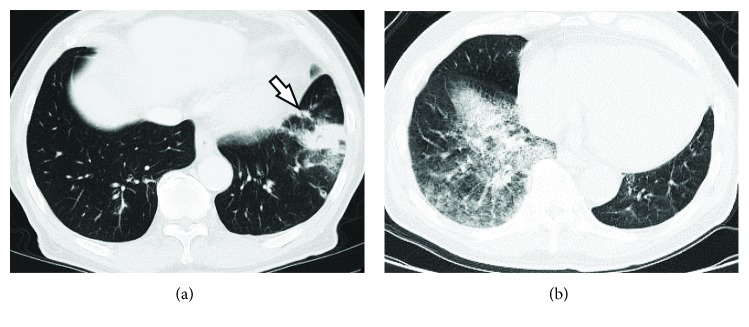
Axial computed tomography (CT) images. (a) A follow-up chest CT image obtained before left lobectomy shows a solid mass accompanied by infiltrative and reticular shadows, and the shadow of a suspected skip lesion (arrow) is also found near the mass. (b) Chest CT image obtained 1 month before partial resection of the right lung shows a widespread infiltrative shadow.

The subject of this case study provided written informed consent for participation. A 75-year-old male with a 50 pack-year smoking history presented for evaluation of abnormal findings on chest computed tomography (CT). Chest CT demonstrated a poorly demarcated, 4 cm-diameter mass with air bronchogram in the left lateral basal lung. The solid mass was accompanied by infiltrative and reticular shadows. Enlargement of hilar or mediastinal lymph nodes and organ metastasis were not seen. A follow-up chest CT two months later showed expansion of the mass, and a shadow suspected to be a skip lesion was also found near the mass (Figure 1(a)). Because transbronchial lung biopsy revealed AC, we performed a thoracoscopic lobectomy. The patient received 10 cycles of adjuvant chemotherapy with paclitaxel and carboplatin 2 months after surgery. No local recurrence or metastasis was observed on a chest CT scan obtained 12 months after surgery. However, a chest CT scan obtained 20 months after the last course of chemotherapy showed a widespread infiltrative shadow in the right posterior basal lung. Moreover, the shadow gradually expanded over the course of 1 year (Figure 1(b)). No metastatic lesion was detected in the left lung and extrapulmonary organs. Sputum cytology detected AC, and we completed a thoracoscopic partial resection. No adjuvant therapies were given. The patient's postoperative course was uneventful, and there were no signs of recurrence or metastasis, even after 1 year.

### 2.2. Routine Pathological Findings

**Figure 2:**
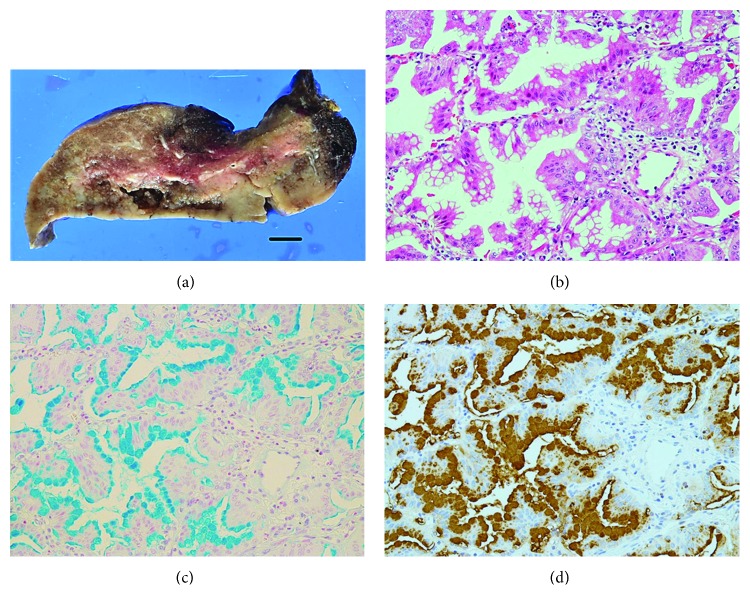
Left lung cancer. (a) Gross view shows an ill-defined gelatinous tumor with a diffuse pneumonia-like consolidation. Bar, 1 cm. (b) Columnar tumor cells with intracytoplasmic vacuoles reveal a papillary growth pattern. H&E stain, ×200. (c) Multiple mucin-producing tumor cells. Alcian blue stain, ×200. (d) Several tumor cells are positive for MUC 5AC. MUC 5AC immunostain, ×200.

Both resected tumors were nearly macro- and microscopically identical. Macroscopically, both tumors were ill-defined and gelatinous with a diffuse pneumonia-like consolidation (Figure 2(a)). Histopathologically, the majority of each tumor exhibited lepidic or papillary growth consisting of columnar tumor cells with varying amounts of intracytoplasmic mucins (Figures 2(b) and 2(c)). Nonmucinous component was seen to the same extent (approximately 20%) in both tumors. Invasive foci, with a maximum diameter of 2 cm, were found in both tumors; however, neither lymphatic nor vascular permeation was detected. Furthermore, no pleural invasion was seen. Once detached from the main tumor, innumerable isolated lepidic or papillary lesions considered as AM were observed in both tumors.

### 2.3. Immunohistochemistry

Immunohistochemical analyses were performed on serial sections prepared from 20% buffered formalin-fixed, paraffin-embedded tissues and carried out with Leica Bond-Max (Leica Biosystems, Australia).

#### 2.3.1. Immunostaining for ANX A2, MUC 5AC, and TTF-1

Mouse anti-human ANX A2 antibody (clone 5/Annexin II, BD Transduction Laboratories, USA) [15], mouse anti-human MUC 5AC antibody (clone 45M1, Thermo Fisher Scientific, USA), and mouse anti-human TTF-1 antibody (clone SP141, Biocare Medical, USA) were used as the primary antibodies. The sections were subjected to heat antigen retrieval with a citrate-based solution (pH 6.0) for 30 min (ANX A2 staining) or EDTA-based solution (pH 9.0) for 20 min (MUC 5AC staining and TTF-1 staining).

Endogenous peroxidase was blocked with 3% hydrogen peroxide for 5 min. The primary antibodies were applied to the sections at dilution ratios of 1/2000, 1/100, and 1/100. Reaction products were visualized with 3,3′-diaminobenzidine. For negative control of anti-ANX A2 antibody, the antibody absorbed by an excessive amount of recombinant ANX A2 or 0.01 M phosphate-buffered saline was used instead of the primary antibody. No apparent immunoreactivity was found in the negative control sections.

**Figure 3:**
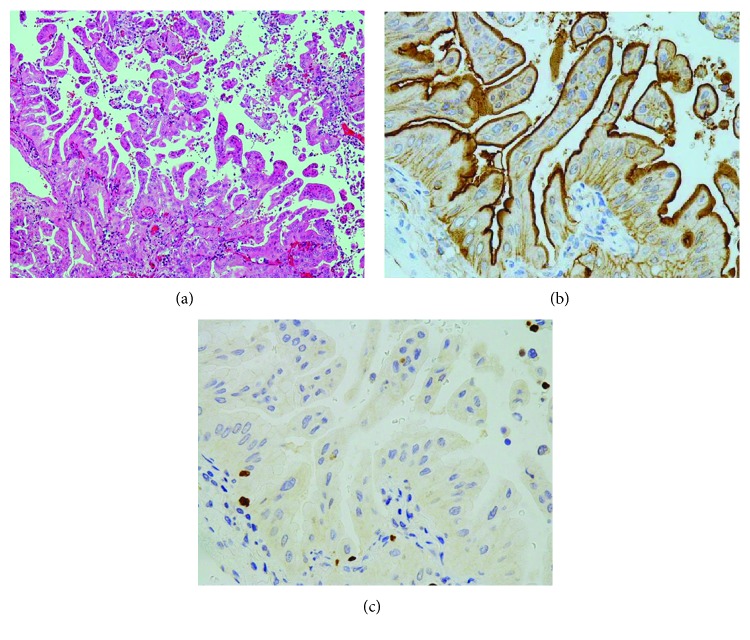
Detachment of tumor cells in the primary site of left lung cancer. (a) Villous-shaped tumor cells project and separate into the alveolar space. H&E stain, ×100. (b) Immunopositivity on the cell-cell borders is weakened as tumor cells project or separate. Annexin A2 immunostain, ×400. (c) Tumor cells are negative, whereas the remaining alveolar epithelial cells are positive. TTF-1 immunostain, ×400.

**Figure 4:**
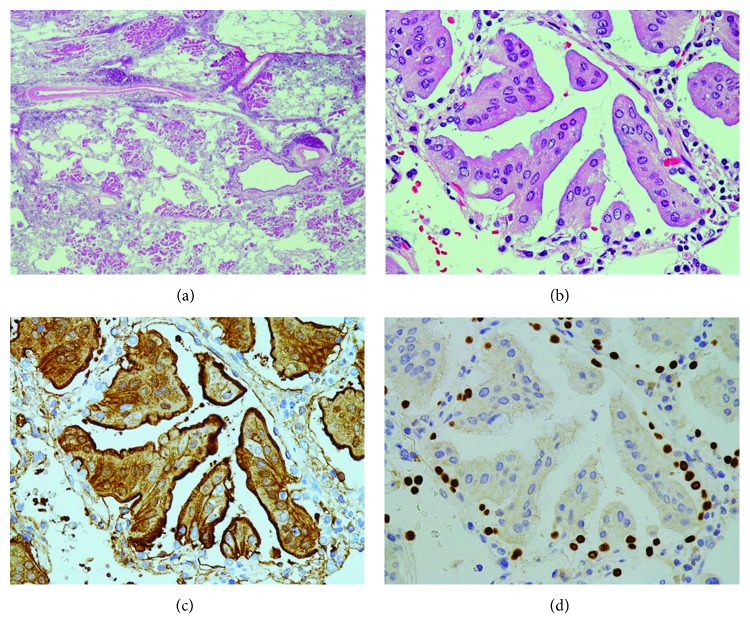
Aerogenous metastasis of left lung cancer I. (a) Many isolated lesions are seen. H&E stain, ×40. (b) Small tumor cell aggregates are attached to alveolar epithelial cells, forming a protrusion. Some of the alveolar epithelial cells have an obscure nucleus and are detached within the alveolar spaces. H&E stain, ×400. (c) Tumor cell aggregates represent the immunopositivity near the cell surface with granular cytoplasmic positivity. Alveolar epithelial cells also show a weak positivity near the cell surface. Annexin A2 immunostain, ×400. (d) Alveolar epithelial cells are positive. The arrangement of these cells is well kept; however, some show weakened immunopositivity. TTF-1 immunostain, ×400.

**Figure 5:**
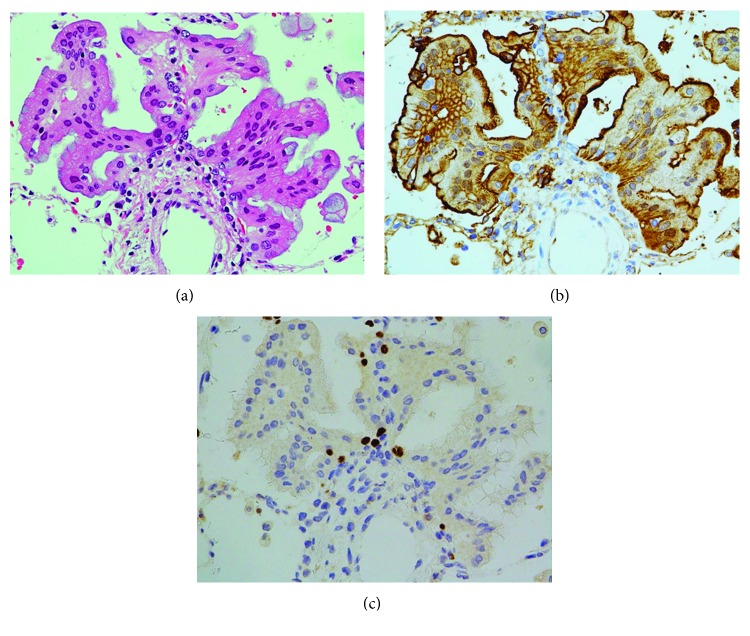
Aerogenous metastasis of left lung cancer II. (a) Tumor cell aggregates adhere to the alveolar wall, and pseudoluminal gaps are seen inside the aggregates. H&E stain, ×400. (b) Cytoplasmic-positive products accumulate in the lateral sides, especially on the underside, forming membranous positivity. Annexin A2 immunostain, ×400. (c) Many alveolar epithelial cells disappear or are indistinct compared with those observed in Figure 4(d). TTF-1 immunostain, ×400.

Immunohistochemical results for ANX A2 were nearly identical in both tumors, with the details noted in the following section. Multiple tumor cells were positive for MUC 5AC (Figure 2(d)). The tumor cells were negative for TTF-1, whereas alveolar epithelial cells (AECs) were positive (Figures 3(c), 4(d), and 5(c)).

#### 2.3.2. Other Immunohistochemical Results

Cells from both tumors were positive for CK7 (clone OV-TL 12/30, DAKO, USA) and MUC 1 (clone Ma695, Leica Microsystems, Germany) and negative for CK20 (clone Ks20.8, DAKO), Napsin A (clone IP64, Leica Microsystems), CDX2 (clone DAK-CDX2, DAKO), MUC 2 (clone Ccp58, Leica Microsystems), and ALK (clone 5A4, Abcam, UK) (data not shown, respectively).

### 2.4. ANX A2 Immunohistochemical Analysis in AM

#### 2.4.1. Primary Lesion

The results are shown in Figures 3(a)–3(c) (Figure 3(b) shows ANX A2 staining). A distinct positivity was found near the tumor cell surface. In the tumor cells with abundant intracytoplasmic mucins, a strong positive reaction was also seen in the mucus part. Furthermore, cell-cell border positivity, especially on the underside, was observed. Cytoplasmic positivity was very fine. The immunoreactivity on the cell-cell borders weakened as the villous-shaped tumor cells projected into the alveolar spaces; however, the positivity near the cell surface was unchanged. Small tumor cell aggregates detached within the alveolar spaces, showing a similar immunoreactivity to that of protrusive tumor cells. The granular cytoplasmic positivity increased in invasive components (data not shown).

#### 2.4.2. AM

**Figure 6:**
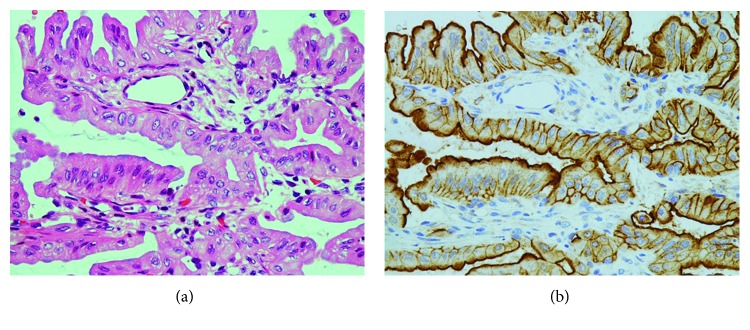
Aerogenous metastasis of left lung cancer III. (a) Tumor cells arrange in one layer along the alveolar wall, and intracytoplasmic mucins are localized on the luminal sides. H&E stain, ×400. (b) Distinct membranous positivity on the cell-cell borders is seen. Annexin A2 immunostain, ×400.

The results are shown in Figures 4(a)–4(d), 5(a)–5(c), and 6(a) and 6(b) (Figures 4(c), 5(b), and 6(b) show ANX A2 staining). Small tumor cell aggregates that were positive near the cell surface and within the cytoplasm were attached to AECs and surrounded them, forming a protrusion (Figure 4(c)). Cytoplasmic positivity of those aggregates was increased compared with that of detached components (Figure 3(b)) of the primary lesion (Figure 4(c)). AECs also showed weak positivity near the cell surface (Figure 4(c)). In the AECs surrounded by tumor cell aggregates, both obscure nuclei and the weakened positivity for TTF-1 were observed (Figure 4). Furthermore, some AECs separated into the air spaces (Figure 4). Small tumor cell aggregates adhered to the alveolar wall where AECs disappeared, and pseudoluminal gaps were scattered inside the aggregates (Figure 5). At this stage, cytoplasmic-positive products accumulated on the lateral sides, especially the underside (Figure 5(b)). After that, as the tumor cells formed one layer along alveolar wall, the membranous positivity on the cell-cell borders became more distinct (Figure 6(b)). In this process, intracytoplasmic mucins were translocated on the luminal sides (Figure 6(a)). The positivity near the tumor cell surface was always found in the above conditions.

## 3. Discussion

In AM, tumor cell aggregates must spread within the alveolar spaces to adhere to AECs, to eliminate them and to stick to the alveolar wall [1]. We predicted that ANX A2, located near the cell surface of both tumor cells and AECs, contributed to cell-cell contacts and elimination of AECs by phagocytosis or separation from the alveolar wall. ANX A2 also plays a role in plasminogen activator-dependent fibrinolysis [5], the degradation of the extracellular matrix (ECM) [5], and metalloprotease activity [9, 10]. ANX A2 likely dissolves the basement membrane of the alveolar wall to facilitate tumor cell binding.

The tumor cells also expressed MUC 5AC. It was reported previously that MUC 5AC promotes cell-ECM adhesion in various cancers [16] and that ANX A2 induces MUC 5AC secretion [17]. MUC 5AC is presumably also involved in adhesion to AECs and/or alveolar wall.

In this case, we observed morphological changes of tumor cell aggregates.

Pseudoluminal gaps might derive from aggregate fusion or separation. In this process, irregularly arranged mucins were translocated to the luminal sides, suggesting restoration of cell polarity [18]. The translocation of cytoplasmic ANX A2 to cell-cell borders may be related to changes in cell morphology, adhesion between tumor cells, and cell polarity restoration. Conversely, the separation of tumor cell aggregates in the primary site may be caused by the degradation of cell-cell adhesions. This is consistent with the attenuation of ANX A2 immunopositivity on the cell-cell borders.

Recent reports have suggested that ANX A2 contributes to cancer invasion. It is derived from ECM degradation and from the promotion of the epithelial-mesenchymal transition (EMT) [19, 20]. STAS/AM is a different progression style than stromal invasion [1–4]; however, it also begins with the detachment of cancer cells [3, 4, 21]. STAS/AM might resemble EMT in terms of reduced cell adhesion. ANX A2 might be involved in decreasing cell-cell contacts in cancer progression.

Indistinct ANX A2-immunoreactivity, in contact with the alveolar wall, may represent poor stromal invasion capacity [12] and weakness of cell-ECM adhesion in IMAC [21]. The same is also thought to be true for the invasive components of these tumors [12].

It has been previously reported that AM in goblet cell-type IMAC originates during tumor cell detachment from the basement membrane of the alveolar wall [21, 22], unlike our observations in the present case. The tumors we used were subdivided into mixed invasive mucinous and nonmucinous AC, and the tumor cells exhibited nongoblet cell morphologies [2].

It is debatable whether the right lung tumor originated due to AM from the left lung tumor or as a second primary lesion [1, 23, 24]. We consider that tumor recurrence 20 months after successful treatment as AM from contralateral lung tumor is an unusual course [25–27]. Furthermore, we have not genetically proved the monoclonality between the two tumors. Considering the clinical course, both large tumors are likely to be double primary lesions with multiple microscopic AMs in the ipsilateral lung.

The present report had some limitations. We analyzed one patient only and lacked a functional assay. However, to our knowledge, ANX A2 expression in the cancer progression to free space, unlike stromal invasion, has not been reported yet. Further investigations are needed to confirm our suppositions.
